# Gastric adenocarcinoma: A one-of-a-kind etiology of autoimmune paraneoplastic encephalitis

**DOI:** 10.1016/j.ijscr.2022.107849

**Published:** 2022-12-26

**Authors:** Luisa Trujillo-Guerrero, Sebastian Vásquez-Garcia, Camilo Ramírez-Giraldo

**Affiliations:** a Universidad del Rosario, Bogotá, Colombia; b Neurologist – Universidad del Rosario, Bogotá, Colombia; c General Surgeon - Hospital Universitario Mayor – Méderi, Bogotá, Colombia

**Keywords:** Paraneoplastic neurological syndrome, Limbic encephalitis, Gastric cancer, Case report

## Abstract

**Introduction and importance:**

Paraneoplastic neurologic syndromes are a group of neurologic disorders that can affect any part of the nervous system and are mediated by immune pathogens produced by cancer. These disorders occur distant to a malignant tumor and are not caused by metastasis, nutritional disorders or side effects of therapy related to the tumor.

**Clinical findings:**

We present the case of a 47-year-old male patient who was admitted to the emergency department due to 1 month of neurological impairment including generalized tonic-clonic movements. He was admitted to the institution and was taken to multiple neurologic tests, all of which were normal, including a negative panel for onconeural antibodies. He persisted with seizures and was taken to a 24-hour video electroencephalogram which showed features consistent with moderate encephalopathy and focal epileptiform activity, which evolved into status epilepticus. Suspecting immune – mediated encephalitis, a therapeutic trial was started with methylprednisolone and plasma exchange, and a positron emission tomography was indicated. The positron emission tomography showed in the brain regions of marked hypometabolism and hypermetabolic thickening of gastric infiltrative aspect fundocorporal topography. Upper gastrointestinal endoscopy revealed in the subcardial region a mass-like lesion with an ulcer-infiltrative appearance, pathology showed an adenocarcinoma.

**Conclusion:**

Autoimmune encephalitis as a paraneoplastic neurological syndrome of a gastric adenocarcinoma have been documented in few patients in the literature. It is important to describe and recognize clinical findings in this cases to be able to suspect malignancy and thus have early diagnosis, start treatment promptly and avoid irreversible neurological sequelae.

## Highlights

• Paraneoplastic neurologic syndromes are neurologic disorders mediated by pathogens produced by cancer.
• Paraneoplastic neurologic syndromes related to solid tumors have an incidence of less than 1%.
• High risk phenotypes are present with neurological manifestations such as limbic encephalitis.
• It is important to describe and recognize PNSs cases, then suspect malignancy and thus have an early diagnosis and start treatment.

## 1 Introduction

Paraneoplastic neurologic syndromes (PNSs) a group of neurologic disorders that affect any part of the nervous system, effects mediated by immune pathogens produced by cancer and develop in 1 of 300 patients with cancer. These disorders occur distant to a malignant tumor and are not caused by metastasis, nutritional disorders or side effects of therapy related to the tumor [1], [2].

In 2004 there were described as standard diagnostic criteria for PNS, they were commonly used and was standardized in this topic, but with the pass of time and advances in the field (new intraneuronal proteins as targets of autoantibodies and pathogenic antibodies against neuronal surface antigens) was identified a need to update this definition. That is why in 2019, a group of experts revised the diagnostic criteria to give as a consensus a clinical scoring system: PNSs-Care score (Table 1), this would lead to simplify clinical decision and define epidemiologic aspects. They defined PNS as possible, probable, and definite in relation with the clinical phenotype, antibody, and cancer, giving a score to each variable with 8 points as definite, 6–7 points probable, 4–5 points possible and <3 points non-PNSs [1].

**Table 1:** PNS-Care Score [1].

		Points
Clinical level	High-risk phenotypes	3
	Intermediate-risk phenotypes	2
	Defined phenotype epidemiologically not associated with cancer	0
Laboratory level	High-risk antibody (>70 % cancer association)	3
	Intermediate risk antibody (30 %–70 %)	2
	Lower risk antibody (<30 %) or negative	0
Cancer	Found, consistent with phenotype and (if present) antibody, or not consistent but antigen expression demonstrated	4
	Not found (or not consistent) but follow-up <2 y	1
	Not found and follow-up ≥2 y	0

PNSs are caused by different mechanisms than side effects of cancer therapy, tumor infiltration or nutritional deficits [3]. As described previously, the incidence is low, except for small-cell lung cancer and thymoma with rates close to 15 %, being for other solid tumors <1 % [3]. We report a case of a patient with an autoimmune encephalitis as a PNSs of a gastric low grade-moderarte differentiated intestinal type adenocarcinoma.

We present the case according to the SCARE guidelines [4].

## 2 Case presentation

We present the case of a previously healthy 47-year-old male patient, with no clinical record, admitted to the emergency department due to 1 month of cognitive impairment (amnesic and attentive changes), behavioral changes, aphasia, and dysarthria, exacerbated by symptoms of severe headache and generalized tonic-clonic movements without associated symptoms. He was admitted to the institution by primary health care and suspecting acute cerebrovascular disease, he was taken a brain computed tomography (CT) scan. Neurology and neuropsychology considered a major neurocognitive disorder in the moderate – severe range, with clinical and pshychometric findings that show a profile of cortico – subcortical impairment; brain CT scan was normal and blood test results were: Antinuclear antibody (ANA) titer: negative, anti-SS-B (LA) antibody 2.48UI (neg), anti-SS-A (RO) antibody 2.24 (neg), anti (SM) antibody 1.81 (neg) and anti NMDA (neg). Cerebrospinal fluid (CSF) examination revealed glucose of 65.94 mg/dl, without pleocytosis, protein 10.31 mg/dl, culture was negative. Brain magnetic resonance imaging (MRI) did not reveal any relevant findings.

**Fig. 1:**
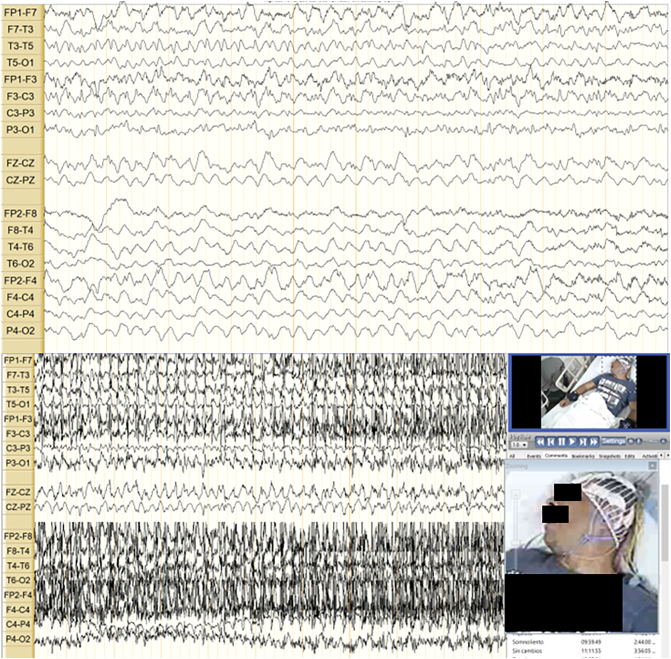
Video EEG: Above: Rhythmic delta activity starting in the left hemisphere and rapidly propagating to the contralateral hemisphere. Below: Electroclinical seizure with spatio-temporal evolution and extended length (not shown here) that fulfills criteria for focal-onset evolving into bilateral convulsive status epilepticus.

Since the patient persisted with seizures, he was taken to a 24-hour video electroncephalogram (Video-EEG) which concluded a moderately expressed encephalopathy, focal epilepsy and status epilepticus being seizure-free in the last 16 h (Fig. 1). Given the persistence of symptoms (clinical status epilepticus, psychomotor agitation, and psychotic symptoms), two normal lumbar punctures and a high suspicion of immune - mediated encephalitis, neurology decided to expand studies with CT of the abdomen and chest and a positron emission tomography (PET-CT). Due to the clinical status of the patient, the persistence of neurological alterations and seizures, a therapeutic test with methylprednisolone 500 mg BID for 5 days and 5 sessions of plasma exchange was made, having improvement in the neurological state of the patient, and achieving absence of seizures.

**Fig. 2:**
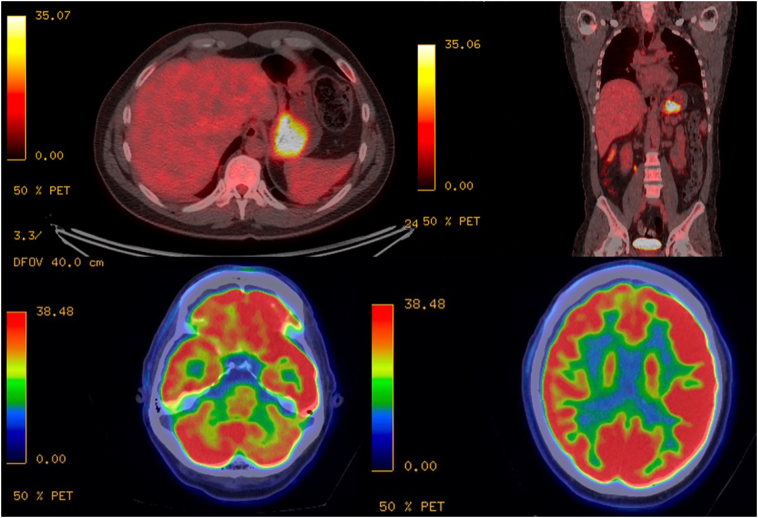
PET-CT: Above: Hypermetabolism thickening of gastric infiltrative aspect fundocorporal topography accompanied by perigastric adenopathies. Below: Brain hypometabolism, diffusely compromising the right cerebral hemisphere, frontal, occipital, parietal and temporal lobes, generating asymmetry with respect to the left side, accompanied by crossed cerebellar diaschisis with diffuse left cerebellar hypometabolism, diffuse right thalamic hypometabolism and relative sparing of the basal ganglia nuclei, some patches of mild hypometabolism in the frontal region.

The abdominal and thorax CT didn't show any relevant findings, but PET-CT (Fig. 2) showed in the brain regions of marked hypometabolism and hypermetabolism thickening of gastric infiltrative aspect fundocorporal topography accompanied by perigastric adenopathies.

**Fig. 3:**
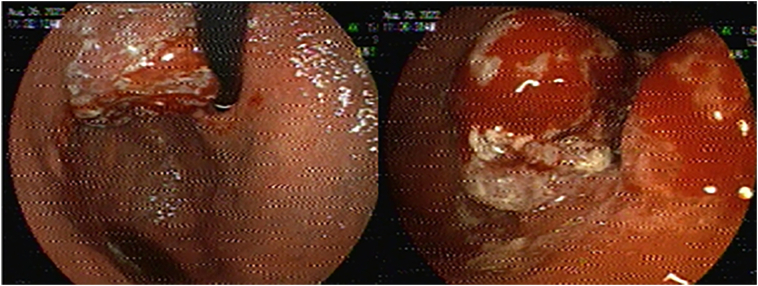
UGE, gastric tumor in the subcardial region.

**Fig. 4:**
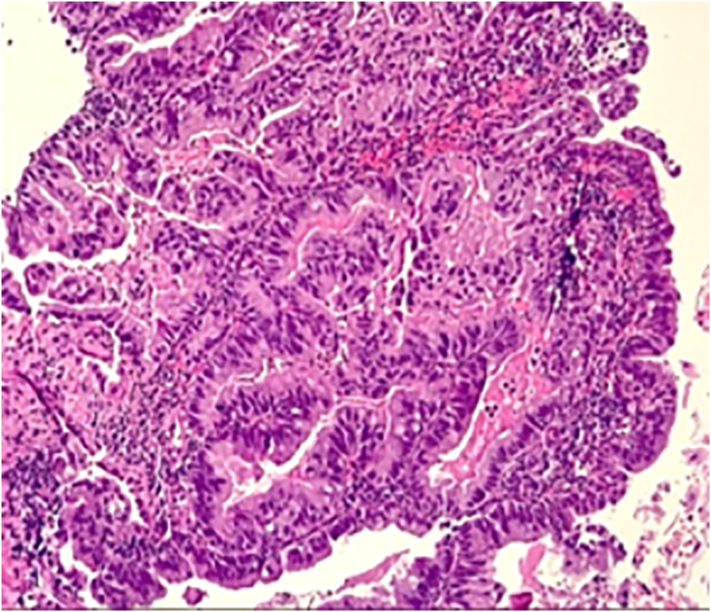
Hematoxylin–Eosin double stain, histologically, the lesion shows a low grade- moderarte differentiated intestinal type adenocarcinoma.

Upper gastrointestinal endoscopy (UGE) (Fig. 3) revealed in the subcardial region, including the gastric body, a mass-like lesion with an ulcer-infiltrative appearance highly suggestive of malignancy. Pathology (Fig. 4) of this lesion showed a low-grade/moderate differentiated intestinal type adenocarcinoma, *Helicobacter pylori*: negative, HER-2: negative, immunohistochemical studies show reactivity in tumor nuclei with MSH2, MSH6, MLH 1 and PMS2.

Based on the anatomopathological examination, the final diagnosis was T3N1M0 Stage III fundocorporal gastric adenocarcinoma, according to the Japanese Classification of Gastric Carcinoma and an oncology assessment was indicated for the initiation plan of neoadjuvant management according to his functional status.

## 3 Discussion

PNSs are a heterogeneous group of neurological disorders in patients with malignant tumors [2], [3]. Its incidence varies, being <1 % in patients with solid tumors, such as the case presented above, a gastric adenocarcinoma [2], [5]. The studies carried out showed that the symptoms of the patient were not associated with a primary neurological pathology per se, making it necessary to perform plasma exchange as a therapeutic trial to control the neurological symptoms presented given the suspicion of an autoimmune-mediated encephalitis.

According to the diagnostic criteria of 2004 described by Graus et al., the classic PNS, currently known as “high-risk phenotypes” present with neurological manifestations: encephalomyelitis, limbic encephalitis, subacute cerebellar degeneration (PCD), opsoclonus/myoclonus syndrome (OMS), subacute sensory neuropathy, chronic gastrointestinal pseudo-obstruction, Lambert-Eaton myasthenic syndrome (LEMS) and dermatomyositis, which are associated with tumors and onconeural antibodies [1], [2], [6]. In this case, no antibody was detected, maybe because the tested panel didn't include the specific onconeural autoantibody involved, or, because it constitutes another less-described molecule that needs other specific test. However it was described as an autoimmune encephalitis, a high-risk phenotype (previous classic PNSs) with a PNS-Care Score of 7 points making it a probable diagnostic level, also, it didn't imply the presence of onconeural antibodies [1], [6]. In our patient, the neurological impairments were evident 4 weeks before the detection of gastric cancer, removal of antigens by plasma exchange made symptoms reversible, despite that it has been reported that PNSs is unresponsive to treatment when the deficits are fully established [3].

One of the high-risk phenotype is the limbic encephalitis [7], [8] described as short-term memory loss, psychiatric manifestations, seizures, symptoms that rapidly progress most of the time in <3 months [1], [6]. There are some antibodies associated such as gamma-aminobutyric-acid B receptor (GABABR) and α-amino-3-hydroxy-5-methyl- 4-isoxazolepropionic acid receptor (AMPAR) present in almost 50 % of the cases [6], and with the study of this onconeural antibodies, it has been established that the development of PNSs is immune-mediated [9]; however, these tests were not available in our institution. With the clinical manifestations in the patient, findings on studies, and the resolution of symptoms after plasma exchange, we can conclude that neurological symptoms were paraneoplastic [10]. Supporting these findings, Takashi, et al. in 2012, made a review of the literature and exposed 10 cases of gastric cancer presented as PNSs and found common characteristics, one related to our case: men and neurological impairment prior the diagnostic of gastric malignancy, and other less related with the case: older patients and neuroendocrine component or predominance and a better oncological outcome for patients with PNSs [10]. These give us an idea of differential diagnosis with patients with neurological symptoms where a primary neurological disease is not evident with the initial approach.

These neurological manifestations are scarce in the literature, considering these “high-risk phenotypes”, we found a series of 24 patients with opsoclonus - myoclonus syndrome, where 10 were idiopathic and 14 paraneoplastic and one of them had a gastric adenocarcinoma. Within the features described in those of the group of PNSs, unlike the idiopathic ones; they were older, with an average age of 66 years, and had a higher frequency of encephalopathy. This patient with gastric cancer, was a 58-year-old female, with symptoms that started one month previous, she was taken to immunotherapy with steroids without response and she died due to encephalopathy 6 months later with a post-mortem study that showed a local infiltrating gastric adenocarcinoma [11]. Another case of a high-risk phenotype with gastric adenocarcinoma is described by Meglic et al.; a 73-year-old male with PCD with anti-Yo antibodies in serum a CSF. In their review they found this case new, with no previous reports of gastric adenocarcinoma associated with PCD and anti-Yo antibodies [12].

Biotti, et al., described that these PNSs in gastric cancer are rare, and when they are present, they are associated with progressive neuropathy, cerebellar ataxia, or Lambert–Eaton myasthenic syndrome [13]. With the low incidence of PNSs in gastric tumors, there are few cases reported in the literature, with adenocarcinomas being limited [1], [2], [3], [4], [5], [6], [7]. In 2016, Uneno et al., carried out a case report and literature review of a patient with HER-2 positive gastric cancer (adenocarcinoma), where they associated oncological management with good oncological and neurological results [9]. In this review, they documented 11 cases of patients with gastric cancer with onconeural antibodies in eight of them. In total, until 2016, there were described 12 cases, 75 % had antibodies recognized [9]. In this review, in addition to cases described by Uneno, they reported 4 more of PNSs, with concomitant gastric cancer, in these new cases there were onconeural antibodies in 2 patients [2]. These studies and what is reviewed and described in this paper show in the literature a total of 18 cases (counting ours) of PNSs associated with a malignant lesion of the stomach, with the presence of onconeural antibodies in 61.1 % (11 patients) [1], [9], [14]. In Table 2, we summarize the cases found in the literature that we previously described. With this in mind, it is possible to find an average age of presentation of 63.6-years-old, being more common in males (75 %).

**Table 2:** PNSs in gastric cancer.

Age	Gender	Gastric cancer type	Antibody	Neurologic presentation
58	Male	Neuroendocrine	N/A	Paraneoplastic cerebellar degeneration
63	Male	Neuroendocrine	Anti-Ri antibody	Paraneoplastic cerebellar degeneration
59	Male	N/A	N/A	Opsoclonus–myoclonus
73	Male	Poorly	Anti-Yo antibody	Paraneoplastic cerebellar degeneration
64	Male	Neuroendocrine	N/A	Paraneoplastic limbic encephalitis
77	Male	N/A	Glur	Paraneoplastic limbic encephalitis
71	Male	N/A	Anti-Yo antibody	Paraneoplastic cerebellar degeneration
72	Female	Signet-ring cell	N/A	Subacute sensory neuropathy, systematic myositis
63	Female	Neuroendocrine moderate	Anti-Hu antibody	Subacute sensory neuropathy
72	Male	Neuroendocrine	N-type VGCC	Paraneoplastic limbic encephalitis
61	Male	Adenocarcinoma	Anti-Ma antibody	Paraneoplastic limbic encephalitis
38	Female	Neuroendocrine	N/A	Paraneoplastic limbic encephalitis
59	Male	Adenocarcinoma	N/A	Opsoclonus–myoclonus
71	Male	Adenocarcinoma	Anti-Hu antibody	Paraneoplastic limbic encephalitis
70	Female	Adenocarcinoma	N/A	Lambert–Eaton myasthenic syndrome
N/A	N/A	N/A	Anti-Hu antibody	Encephalomyelitis
N/A	N/A	N/A	Anti-Hu antibody	Encephalomyelitis
47	Male	Adenocarcinoma	N/A	Paraneoplastic limbic encephalitis

Gastric cancer can cause unpleasant and sometimes harmful symptoms in patients, such as bleeding, obstruction, pain, nausea or vomiting [15]. However, there are atypical manifestations in the context of SNP such as different neurological entities; entities that can precede the diagnosis of the tumor weeks or months. Proper recognition and management of these patients is important to achieve, as in our case a total improvement in neurological symptoms having no irreversible damage. Encephalitis as a SNP of a gastric adenocarcinoma has been documented in few patients in the literature. Its approach is a challenge for the physician, but nevertheless its early detection is the key to achieve adequate oncological and neurological results.

## Abbreviations

PNSs: paraneoplastic neurologic syndromes
CSF: cerebrospinal fluid
MRI: brain magnetic resonance imaging
Video-EEG: video electroncephalogram
CT: computed tomography
PET-CT: positron emission tomography
UGE: upper gastrointestinal endoscopy
PCD: subacute cerebellar degeneration
OMS: opsoclonus/myoclonus syndrome
LEMS: Lambert-Eaton myasthenic syndrome
GABABR: gamma-aminobutyric-acid B receptor
AMPAR: α-amino-3-hydroxy-5-methyl-4-isoxazolepropionic acid receptor

## Consent

Written informed consent was obtained from the patient for publication of this case report and accompanying images. A copy of the written consent is available for review by the Editor-in-Chief of this journal on request.

## Ethical approval

Ethical approval of institutional committee was made previous publication (CEI-UR 2104-CV1621).

## Funding

This research did not receive any specific grant from funding agencies in the public, commercial, or not-for-profit sectors.

## Author contribution

Luisa Fernanda Trujillo-Guerrero: Make substantial contributions to conception and design, acquisition of data, analysis and interpretation of data.

Sebastian Vásquez-Garcia: Participate in drafting the article and revising it critically for important intellectual content.

Camilo Ramírez-Giraldo: Participate in drafting the article and revising it critically for important intellectual content.

## Guarantor

Camilo Ramírez-Giraldo.

## Registration of research studies

Not applicable.

## Provenance and peer review

Not commissioned, externally peer-reviewed.

## Declaration of competing interest

All authors declare no conflicts of interest.
